# The Prognostic Performance of Ferritin in Patients with Acute Myocardial Infarction: A Systematic Review

**DOI:** 10.3390/diagnostics12020476

**Published:** 2022-02-13

**Authors:** Crischentian Brinza, Mariana Floria, Iolanda Valentina Popa, Alexandru Burlacu

**Affiliations:** 1 Faculty of Medicine, University of Medicine and Pharmacy, 700115 Iasi, Romania; crischentian.brinza@d.umfiasi.ro (C.B.); floria.mariana@umfiasi.ro (M.F.); alexandru.burlacu@umfiasi.ro (A.B.); 2 Institute of Cardiovascular Diseases, 700503 Iasi, Romania; 3 Military Emergency Clinical Hospital, 700483 Iasi, Romania

**Keywords:** ferritin, acute myocardial infarction, adverse outcomes, prognosis, mortality

## Abstract

The potential benefit of ferritin evaluation resides in its association with adverse outcomes in patients with various pathological conditions. We aimed to conduct the first systematic review evaluating the association between ferritin levels and adverse cardiovascular outcomes in patients with acute myocardial infarction (AMI) during short- or long-term follow-up. Seven studies investigating various endpoints (mortality, major adverse cardiovascular events-MACE, the decline of the left ventricular ejection fraction-LVEF, left ventricular aneurysm development-LVA) were included. AMI patients with low or increased ferritin values tended to have higher in-hospital and 30-day mortality rates. Low and high ferritin levels and chronic kidney disease were independently associated with increased risk of LVA formation. High ferritin concentrations were linked to an accentuated LVEF decline in ST-elevation myocardial infarction patients treated by percutaneous coronary intervention. Both low and high ferritin values were also associated with the duration of hospitalization in patients with AMI during hospital stay and at more extended follow-up. Ferritin evaluation represents a simple investigation that could identify high-risk patients with AMI who might benefit from closer monitoring and specific therapeutic interventions. These data should be confirmed in large trials in the context of currently available therapies for heart failure and AMI.

## 1. Introduction

Ferritin represents an essential protein in iron metabolism, reflecting body iron homeostasis and a biomarker of the inflammation process. Iron status and acute or chronic inflammation are involved in controlling ferritin levels [1]. The potential benefit of ferritin evaluation resides in its association with adverse outcomes in the general population and patients with various pathological conditions. In this regard, both low and high ferritin values were associated with all-cause mortality in some general population studies. In a Danish population study (enrolling 8988 participants with a long-term follow-up-median 23 years), a significantly higher rate of all-cause and cardiovascular mortality in participants with increased ferritin levels (≥200 µg/L) was observed [2]. In the English longitudinal study of aging, women with the lowest ferritin levels exhibited a higher risk of all-cause mortality during a 7.7-year follow-up period. In the case of male participants without chronic diseases, increased all-cause mortality was recorded in the group with the highest ferritin levels, highlighting a possible gender-specific risk stratification [3].

Ferritin could also be used as a prognostic marker in patients with chronic diseases, such as chronic kidney disease. In a trial involving patients with end-stage kidney disease and haemodialysis, markedly increased ferritin levels (>1500 ng/mL) were linked to higher all-cause mortality during five years of follow-up [4]. In a Japanese registry of dialysis patients, high serum ferritin (>496 ng/mL) was associated not only with all-cause mortality but also with mortality due to cardiovascular and infectious causes. However, the effect was documented only in patients with haemodialysis but not in those with peritoneal dialysis [5].

Besides all-cause and cardiovascular mortality, ferritin was also linked to a higher burden of cardiovascular risk factors, including metabolic syndrome. A recent meta-analysis which analysed 14 studies observed a higher risk of metabolic syndrome in patients with increased serum ferritin [6]. The authors from one study revealed that ferritin levels were correlated independently with coronary artery calcium score, suggesting the potential role of ferritin in the early detection of atherosclerosis [7]. In addition, ferritin could be used to identify patients at greater risk of developing atrial fibrillation, as documented in a meta-analysis [8].

Whether there is a correlation between ferritin levels and the risk of ischemic heart disease is of considerable debate and is extensively studied in the literature. An early trial from 2003 did not observe any correlation between ferritin and coronary heart disease or stroke during long-term follow-up (17 years) [9]. Conversely, in another study, the authors documented a more than five-fold higher risk of acute myocardial infarction (AMI) in patients with ferritin values above 200 µg/L, an effect maintained after multivariate analysis [10].

Concerning the prognostic value of iron deficiency in acute coronary syndromes (ACS), a recent meta-analysis of seven studies recorded a higher risk of adverse outcomes during long-term follow-up in patients with iron deficiency [11]. However, ferritin was rarely used in defining iron deficiency in analysed studies, and this meta-analysis did not focus primarily on patients with AMI.

Thus, we aimed to systematically review the literature and evaluate the association between ferritin levels and adverse cardiovascular outcomes in patients with acute myocardial infarction during short- or long-term follow-up.

## 2. Materials and Methods

We used the updated Preferred Reporting Items for Systematic Reviews and Meta-Analyses (PRISMA) checklist to guide each step of the present systematic review [12].

### 2.1. Data Sources and Search Strategy

In order to obtain eligible studies, we performed a literature search in the following databases: MEDLINE (PubMed), Embase, and Cochrane, without applying language filters. The databases were screened from inception until 1 November 2021. As we investigated the role of ferritin in AMI patients, only studies involving humans were considered in the search process. Moreover, we screened Google Scholar and ClinicalTrials.gov databases and references from relevant studies to retrieve additional clinical studies that could meet the inclusion criteria. The following keywords and MeSH terms were used: “ferritin”, “iron deficiency”, “myocardial infarction”, “acute coronary syndrome”, “percutaneous coronary intervention”, “adverse outcomes”, and “mortality”. The search strategy and terms used for searching in specified databases are provided in Table S1.

### 2.2. Eligibility Criteria and Outcomes

Studies were analysed in the present systematic review if they fulfilled prespecified inclusion criteria: (1) randomized or non-randomized study design; (2) humans aged 18 years or more were included; (3) patients with myocardial infarction, ST-elevation myocardial infarction (STEMI), or non-ST segment elevation myocardial infarction (NSTEMI) were investigated; (4) studies which provided original data concerning the prognostic value of serum ferritin on short- or long-term outcomes, including major adverse cardiovascular events (MACE), mortality, left ventricular ejection fraction (LVEF), heart failure or development of a left ventricular aneurysm. In addition, several exclusion criteria were set: studies available only in abstract, case reports, editorials, overlapping population, unpublished data, conference papers, meta-analyses, and inability to extract data. Two independent investigators who analysed the inclusion and exclusion criteria decided to include a study in the present systematic review. Any disagreements were solved by consensus.

### 2.3. Data Collection

Two independent investigators collected relevant data from included studies: the first author’s name, publication year, design of the study, number of participants evaluated, and their age, clinical setting, reported outcomes, and follow-up period. Afterwards, data were presented as numbers, percentages, odds ratio, the area under the curve (AUC), and *p* values when available.

### 2.4. Quality Assessment

The quality of case-control and cohort studies was assessed using the Newcastle-Ottawa scale. This scale consists of three domains (selection, comparability of groups, investigated outcomes), each of them containing different signalling questions. Stars are assigned for each question if the quality is judged to be high [13]. The quality assessment of studies that did not include a control group of patients was guided by the National Institute of Health (NIH) tool. It encompasses 14 important questions which help to evaluate the quality of a study [14].

## 3. Results

**Figure 1:**
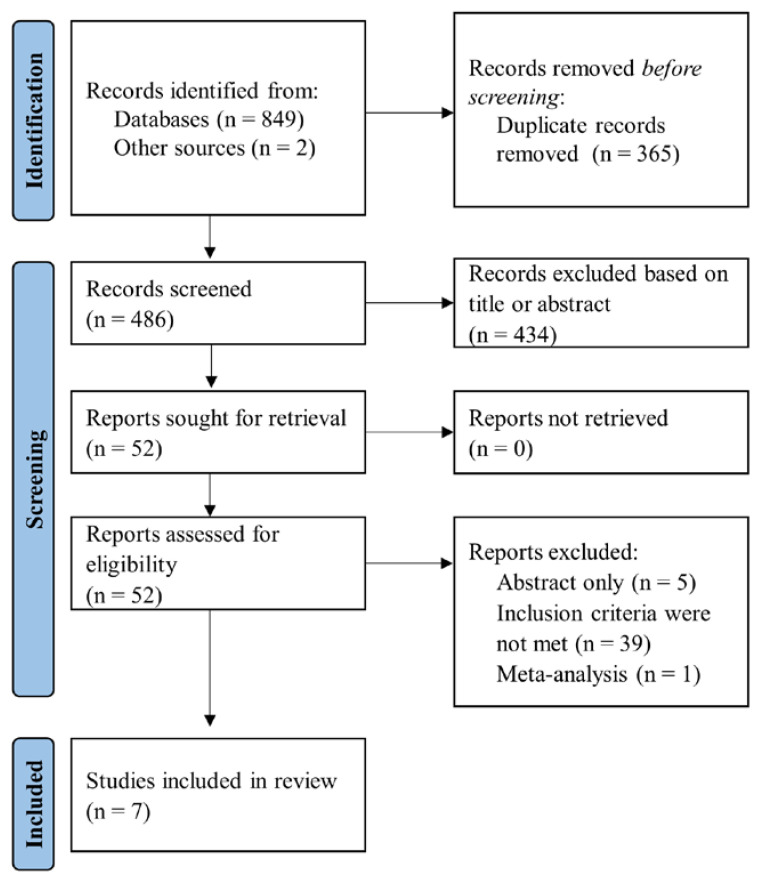
Flow diagram of selected studies in present systematic review.

The databases MEDLINE (PubMed), Embase, and Cochrane were searched using prespecified keywords and retrieved 849 citations. Additional two citations were obtained after screening the Google Scholar database. Initially, we excluded duplicate references and records based on title or abstract screening, leaving 52 studies for eligibility assessment. Two independent investigators assessed the remaining citations’ eligibility criteria and excluded studies that did not fulfil the inclusion criteria. Finally, seven studies were included and analysed in the present systematic review, as reported in the PRISMA flow-chart of selecting studies (Figure 1).

The extracted data from included studies are provided in Table 1 and Table 2 as follows: in the former table, we report general characteristics, design, and demographic data of the studies and population, and in the latter table, we provide data regarding outcomes and results reported in clinical trials.

All investigated studies had a non-randomized observational design [15,16,17,18,19,20,21]. Two studies investigated patients prospectively [16,18], while the cross-sectional design was reported in the other two studies [19,21]. Most of the studies analysed in-hospital adverse events [16,20,21], but the follow-up period varied across studies ranging from 30 days up to 6 months [15,18]. Four studies enrolled patients with STEMI [15,16,18,21], while the rest included patients presenting with all types of AMI [17,19,20].

Two studies evaluated mortality endpoint following AMI as a composite outcome of MACE or in-hospital mortality and Killip class [15,16]. In addition, mortality was also investigated as an individual outcome in the other two studies [20,21]. Dominguez-Rodriguez et al. enrolled patients with STEMI who underwent myocardial revascularization by the primary percutaneous coronary intervention (PCI) [15]. Ferritin values were lower in patients with MACE than in those without MACE at 30-day follow-up (*p* = 0.003). Moreover, low ferritin values were associated with a significantly higher risk of MACE at multivariate analysis (*p* = 0.01), suggesting the possible role of ferritin in identifying high-risk patients. In addition, ferritin had a modest predictive performance for 30-days MACE (AUC = 0.65, *p* = 0.001), with 86% sensitivity and 69% specificity for the established cut-off (83 ng/mL) [15].

Cosentino et al. also investigated patients with STEMI treated by primary PCI and observed discrepant results compared to the study mentioned above [16]. Low ferritin values, as a part of the iron deficiency definition used in the study (ferritin < 100 µg/L or transferrin saturation < 20%), had a protective effect, with a reduced composite outcome of in-hospital mortality and Killip class ≥ 3 (*p* = 0.01). The effect was maintained after adjustment for multiple variables (*p* = 0.02). Moreover, ferritin was correlated with microvascular obstruction (R = 0.17, *p* = 0.04) and high-sensitivity troponin I values (R = −0.29, *p* < 0.001). In addition, a correlation was found between ferritin and mitochondrial dysfunction, defined as cell-free mitochondrial DNA (R = −0.26, *p* < 0.001) [16].

Singh et al. and Malthesh et al. investigated the association between ferritin concentration and in-hospital mortality [20,21]. Patients presenting with STEMI and those with NSTEMI were enrolled in the former study [20]. In-hospital death was recorded more frequently in group of patients with high ferritin values with a cut-off > 220 ng/mL (*n* = 5) when compared to those with low or intermediary ferritin values (respectively, *n* = 1 and *n* = 2), *p* = 0.03 [20]. In the latter study, although ferritin values tended to be higher in patients who died than in survivors, the difference was not statistically significant (*p* = 0.15). However, the sample size was small (*n* = 45), limiting the extrapolation of the results in the case of large cohorts of patients with AMI [21].

Clinical studies also documented an association between ferritin levels and LVEF [18,19,20]. Suzuki et al. enrolled patients with STEMI and successful PCI and measured LVEF at baseline and 6-month follow-up [18]. Patients with high ferritin values (>200 ng/mL) exhibited an accentuated decline in LVEF during follow-up in comparison to those with low ferritin (<100 ng/mL, *p* < 0.01) or intermediary ferritin levels (100–200 ng/mL, *p* < 0.05). However, no differences in LVEF values were recorded at baseline across various ferritin concentrations [18].

In a cross-sectional study, Basu et al. recorded significantly higher ferritin values in patients with LVEF < 35% than in those with LVEF ranging between 35% and 50% (*p* = 0.002) [19]. However, the investigators enrolled only male patients, thus limiting the generalization of the results in all patients. In another trial, Singh et al. observed an increased number of patients with LVEF < 35% in the highest ferritin concentration group (>220 ng/mL) [20]. These data highlight the importance of ferritin for recognizing patients at high risk of cardiac function decline following AMI, but the results should be validated in large clinical studies.

Besides mortality, MACE, and LVEF, the role of ferritin was also studied in other outcomes, similar to left ventricular aneurysm development, recurrent angina, heart failure, and duration of hospitalization. Feng et al. recorded that both, low or high ferritin values, were independently associated with adverse left ventricular remodelling and development of aneurysm (*p* = 0.042) [17]. In addition, Malthesh et al. reported a correlation between ferritin values and duration of hospitalization (*p* = 0.01) in patients presenting with STEMI [18]. Regarding recurrent angina and heart failure, Singh et al. did not record any significant association with serum ferritin levels [20].

The overall quality of included studies was evaluated using the Newcastle–Ottawa scale and NIH tool for observational studies and was judged to be fair to low, as reported in Tables S2 and S3.

## 4. Discussion

As far as we know, the present systematic review is the first one addressing the association between ferritin levels and adverse events, particularly in patients presenting with AMI. Our paper shortens the path to achieving appropriate monitoring and timely therapeutic interventions for high-risk patients with AMI by proving the association between ferritin levels and adverse cardiovascular outcomes during short- or long-term follow-up.

In the last decade, iron metabolism gained interest and was extensively studied in patients with cardiovascular diseases [22]. Markers of iron metabolism alteration (ferritin, transferrin saturation) could be easily evaluated but might have important prognostic and treatment values. A notable benefit of iron metabolism assessment is represented by heart failure patients, as iron deficiency could be linked to increased mortality and hospitalization rates [22]. The 2021 guidelines for the diagnosis and treatment of heart failure endorsed by the European Society of Cardiology (ESC) advocated screening for iron deficiency (including ferritin and transferrin saturation) with class IC recommendation [23]. In addition, heart failure patients with LVEF < 50% should be considered for iron supplementation if iron deficiency is diagnosed (ferritin < 100 ng/mL or ferritin 100–299 ng/mL with transferrin saturation < 20%) [23]. However, the latest ESC guidelines focused on managing patients with STEMI and non-STEMI did not mention the potential prognostic or treatment values of iron deficiency [24,25]. Thus, the role of iron deficiency and particularly ferritin levels in patients presenting with AMI represents a further direction for clinical research.

Ferritin could be regarded as a marker of both iron metabolism and inflammation. Ferritin decreases in absolute iron-deficient states and increases in inflammatory conditions when the iron is reduced due to functional causes [22]. Oxidative stress and promotion of inflammation and atherosclerosis could represent a pathophysiological link between ferritin levels and cardiovascular outcomes following AMI. Some studies documented that serum ferritin enhanced low-density lipoprotein oxidation, which is an established risk factor for atherosclerosis progression and injury of the coronary arteries endothelium [26,27]. 

One study observed that in overweight and obese patients characterized by a pro-inflammatory state, ferritin acted more similar to a marker of inflammation than a marker of iron metabolism [28]. These data could represent a possible explanation for adverse clinical events associated not only with lower ferritin values when it indicates an absolute iron deficiency, but also with increased ferritin values when it indicates an inflammatory condition and subsequent functional iron deficiency [22,28].

Studies investigating serum ferritin in AMI patients included various adverse endpoints: mortality, MACE, the decline of LVEF, left ventricular aneurysm development, recurrent angina, heart failure, and duration of hospitalization. Data regarding the role of ferritin in risk of mortality and MACE stratification were somewhat discrepant; however, studies were heterogeneous [15,16,20,21]. Different outcome definitions and follow-up duration could represent potential sources of discordant results. Hence, one study investigated mortality as a composite outcome of MACE during 30 days of follow-up [15], another study reported in-hospital mortality as a composite of mortality and Killip class [16], while two studies analysed in-hospital mortality as individual endpoints [20,21]. In addition, different population characteristics and clinical settings could also represent a cause of discrepant results. In this regard, one trial enrolled patients with STEMI who underwent primary PCI within 6 h from symptoms onset [15], while the other trials included STEMI patients treated by primary PCI within 12 h from symptoms onset [16] or patients presenting with both, STEMI or non-STEMI [20]. AMI patients with markedly low or increased ferritin values tended to have higher in-hospital and 30-day mortality rates [15,20,21].

Thus, integrating ferritin in existing or future clinical models for risk stratification might increase the prognostic performance, delineating high-risk patients who could benefit from closer monitoring. 

Although iron deficiency was linked to more serious myocardial damage in patients with AMI in one study, the composite outcome of in-hospital mortality and Killip class ≥ 3 was lower [16]. This effect was partly attributed to a decreased reperfusion injury in patients with iron deficiency who underwent primary PCI. Thus, extensive clinical studies are warranted to establish the impact of ferritin on early outcomes after primary PCI and on long-term mortality risk.

Besides mortality risk stratification, ferritin could be used as a risk marker for left ventricular aneurysm development. In one study, low and high ferritin levels and chronic kidney disease were independently associated with increased risk of left ventricular aneurysm formation after multivariate adjustment [17]. The authors used a wider ferritin normal range with a lower cut-off limit (7.0–323 ng/mL). However, results are limited by the observational design and a relatively small number of patients; thus, these data should be confirmed in more extensive and prospective studies.

Moreover, high ferritin concentrations were linked to an accentuated LVEF decline in STEMI patients treated by PCI [18]. Nevertheless, the association between ferritin and LVEF should be evaluated in a contemporary cohort of patients, with currently available guidelines-directed therapies for heart failure patients.

Our paper has several limitations. First, data inhomogeneity hampered the possibility to conduct a meta-analysis, as the included studies had different methods to describe the effect: some reported the odds ratio, others used the area under the curve, while others reported only *p* values, events number or sensitivity and specificity. Thus, the data were not appropriate for pooling. Second, the small size of the population limits the generalizability of the results. Third, no consensus exists on the cut-off ferritin values. Common cut-off values should be established in order to record generalizable outcomes. Fourth, most clinical studies focused on a single ferritin assessment; therefore, serial ferritin measurements could have different correlations with adverse events.

## 5. Conclusions

Both low and high ferritin values were associated with adverse events in patients with AMI during the hospital stay and at more extended follow-up assessment as documented in clinical studies. Among adverse outcomes, ferritin was linked to increased mortality risk, accentuated LVEF decline during follow-up, left ventricular aneurysm formation, and duration of hospitalization. Our study represents a step further to closer monitoring and timely therapeutic interventions for high-risk patients with AMI by assessing the role of a relatively simple investigation, such as ferritin. However, these data should be confirmed in large trials involving contemporary patients in the context of currently available therapies for heart failure and myocardial infarction.

## Figures and Tables

**Table 1:** General characteristics of studies included in present systematic review.

Author, Year	Design	Patients No	Age, Mean/Median ± SD	Setting	Parameters	Outcomes	Follow-Up
Dominguez-Rodriguez et al., 2013	Observational, single centre	258	64 ± 13—patients with MACE	Patients with STEMI treated with primary PCI within 6 h from symptoms onset	Low ferritin concentration (one assessment)	MACE (cardiovascular death, non-fatal myocardial infarction, re-admission for unstable angina)	30-days
			65 ± 12—patients without MACE				
Cosentino et al., 2020	Observational, single centre, prospective	420	65 ± 12	Consecutive patients with STEMI treated with primary PCI	Low ferritin values (<100 µg/L) or transferrin saturation < 20%	Composite of in-hospital mortality and Killip class ≥ 3	In-hospital
Feng et al., 2018	Observational, single centre, case-control	60—cases (AMI with LVA)	67.3 ± 11.8—patients with LVA	Patients with AMI with LVA	Patients were divided in three groups according to ferritin values: group 1 (<7.0 ng/mL), group 2 (7.0–323.0 ng/mL), group 3 (>323.0 ng/mL)	LVA formation	-
		133—controls (AMI without LVA)	66.03 ± 12.83—patients without LVA				
Suzuki et al., 2012	Observational, prospective	53	66	Patients with STEMI with successful PCI within 24 h from symptoms onset	Ferritin levels were stratified in tertiles: <100 ng/mL, 100–200 ng/mL, >200 ng/mL	LVEF at baseline and during follow-up	6 months
Basu et al., 2014	Observational, cross-sectional, single centre	43—patients with AMI	50.7 ± 15.6—patients with AMI	Male patients with AMI	Ferritin concentration	LVEF < 35% vs. LVEF 35–50%	-
		40—patients without AMI	47.9 ± 18.3—patients without AMI				
Singh et al., 2021	Observational	150	-	Patients with STEMI (*n* = 50), NSTEMI (*n* = 50) and healthy controls (*n* = 50)	Ferritin levels were stratified in tertiles: <120 ng/mL, 120–220 ng/mL, >220 ng/mL	(a) LVEF (b) Killip class (c) mortality (d) recurrent angina	In-hospital
Malthesh et al., 2020	Observational, cross-sectional	45	63.4 ± 11.8	Patients with STEMI	Ferritin concentration	(a) duration of hospitalization (b) mortality	In-hospital

AMI, acute myocardial infarction; LVA, left ventricular aneurysm; LVEF, left ventricular ejection fraction; MACE, major adverse cardiovascular events; NSTEMI, non-ST segment elevation myocardial infarction; STEMI, ST-segment elevation myocardial infarction; PCI, percutaneous coronary intervention.

**Table 2:** Results reported in studies analysed in the present systematic review.

Study	Outcomes	Results	
Dominguez-Rodriguez, 2013	MACE at 30-days	*Low ferritin values*	
		OR 1.003 (95% CI, 1.001–1.006)—multivariate analysis	*p* = 0.01
		AUC 0.65 (95% CI, 0.562–0.753)	*p* = 0.001
		Ferritin cut-off 83 ng/mL: sensitivity 86%, specificity 69%	
Cosentino, 2019	Composite of in-hospital mortality and Killip class ≥ 3	*Ferritin < 100 µg/L or transferrin saturation < 20%*	
		Unadjusted OR 0.48 (95% CI, 0.28–0.87)	*p* = 0.01
		Adjusted OR 0.50 (95% CI, 0.27–0.93)	*p* = 0.02
Feng et al., 2018	LVA formation	*Low or high levels of ferritin*	
		OR 1.151 (95% CI, 1.050–1.252)—adjusted for multiple variables	*p* = 0.042
Suzuki et al., 2012	LVEF at baseline	No significant differences were observed across ferritin concentrations	
	LVEF decline during follow-up	LVEF decline was accentuated in patients with ferritin > 200 ng/mL vs. patients with ferritin < 100 ng/mL	*p* < 0.01
		LVEF decline was accentuated in patients with ferritin > 200 ng/mL vs. patients with ferritin 100–200 ng/mL	*p* < 0.05
Basu et al., 2014	LVEF < 35% vs. LVEF 35–50%	Ferritin 211.38 ± 52.66 ng/mL vs. ferritin 167.27 ± 30.05 ng/mL	*p* = 0.002
Singh et al., 2021		*Ferritin < 120 ng/mL vs. ferritin 120–220 ng/mL vs. ferritin > 220 ng/mL*	
	LVEF < 35%	2 patients vs. 4 patients vs. 9 patients	*p* = 0.01
	Recurrent angina	2 patients vs. 10 patients vs. 8 patients	*p* = 0.09
	Heart failure	4 patients vs. 6 patients vs. 8 patients	*p* = 0.1
	Death	1 patient vs. 2 patients vs. 5 patients	*p* = 0.03
Malthesh et al., 2020	Duration of hospitalization	Spearman’s R coefficient 0.38	*p* = 0.01
	Mortality	Ferritin 214.2 ± 157.3 µg/dL (deceased patients) vs. ferritin 126.6 ± 93.0 µg/dL (survivors)	*p* = 0.15

AUC, area under the curve; LVA, left ventricular aneurysm; LVEF, left ventricular ejection fraction; MACE, major adverse cardiovascular events; OR, odds ratio.

## Supplementary Materials

The following are available online at https://www.mdpi.com/article/10.3390/diagnostics12020476/s1, Table S1: Databases and search strategies used in the present systematic review, Table S2: Quality assessment of included studies using Newcastle–Ottawa scale, Table S3: Quality assessment using NIH tool for observational studies.
