# Supplementary material for: The Prognostic Performance of Ferritin in Patients with Acute Myocardial Infarction: A Systematic Review
Source: Diagnostics (Basel). 2022 Feb 13;12(2):476. doi: 10.3390/diagnostics12020476 (PMC8870888; doi:10.3390/diagnostics12020476)
Supplement: Supplementary file 1 [file diagnostics-12-00476-s001.zip › Table S1.pdf]

Table S1. Databases and search strategies used in present systematic review

Database	Coverage	Search run	Records
**MEDLINE**	1946 to present	ferritin AND myocardial infarction	20
		ferritin AND acute coronary syndrome	3
		ferritin AND myocardial infarction AND (adverse outcomes OR mortality)	11
		(ferritin OR iron deficiency) AND (myocardial infarction OR acute coronary syndrome OR percutaneous coronary intervention OR PCI)	294
			Total records = 328
**Embase**	1966 to present	ferritin AND myocardial infarction	74
		ferritin AND acute coronary syndrome	111
		ferritin AND myocardial infarction AND (adverse outcomes OR mortality)	18
		(ferritin OR iron deficiency) AND (myocardial infarction OR acute coronary syndrome OR percutaneous coronary intervention OR PCI)	126
			Total records = 329
**Cochrane library**	1967 to present	ferritin AND myocardial infarction	54
		ferritin AND acute coronary syndrome	18
		ferritin AND myocardial infarction AND (adverse outcomes OR mortality)	32
		(ferritin OR iron deficiency) AND (myocardial infarction OR acute coronary syndrome OR percutaneous coronary intervention OR PCI)	88
			Total records = 192
ALL RECORDS = 849			
