# Supplementary material for: The Prognostic Performance of Ferritin in Patients with Acute Myocardial Infarction: A Systematic Review
Source: Diagnostics (Basel). 2022 Feb 13;12(2):476. doi: 10.3390/diagnostics12020476 (PMC8870888; doi:10.3390/diagnostics12020476)
Supplement: Supplementary file 1 [file diagnostics-12-00476-s001.zip › Table S2.pdf]

Table S2. Quality assessment of included studies using Newcastle-Ottawa scale.

Study	Case definition	Case representativeness	Selection of controls	Definition of controls	Comparability	Exposure ascertainment	Ascertainment methods	Non-response rate	Total
Feng, 2018	*	*	*	*	*	*	*		7
Basu, 2014	*	*	*	*	*	*	*		7
Singh, 2021	*	*	*	*	*	*			6

NA = not applicable.

Good quality: 3 or 4 stars in selection domain AND 1 or 2 stars in comparability domain AND 2 or 3 stars in outcome/exposure domain. Fair quality: 2 stars in selection domain AND 1 or 2 stars in comparability domain AND 2 or 3 stars in outcome/exposure domain. Poor quality: 0 or 1 star in selection domain OR 0 stars in comparability domain OR 0 or 1 stars in outcome/exposure domain.
